# An exceptional oncological enigma: primary renal Ewing sarcoma in a clinical context—a case report

**DOI:** 10.3389/fonc.2025.1602774

**Published:** 2025-09-09

**Authors:** Wei Lun Tan, Iqbal Hussain Rizuana, Bang Rom Lee, Xeng Inn Fam

**Affiliations:** ^1^ Department of Surgery, Faculty of Medicine, Universiti Kebangsaan Malaysia, Kuala Lumpur, Malaysia; ^2^ Department of Radiology, Faculty of Medicine, Universiti Kebangsaan Malaysia, Kuala Lumpur, Malaysia; ^3^ Department of Pathology, Hospital Picaso, Petaling Jaya, Malaysia; ^4^ Urology Unit, Department of Surgery, Faculty of Medicine, Universiti Kebangsaan Malaysia, Kuala Lumpur, Malaysia

**Keywords:** renal Ewing sarcoma, primitive neuroectodermal tumor (PNET), laparoscopic nephrectomy, chemotherapy, case report

## Abstract

Primary renal Ewing sarcoma/primitive neuroectodermal tumor (PNET) is a very rare and aggressive malignant tumor. We report a case of a young adult who presented with painless hematuria and acute urinary retention and was subsequently diagnosed with localized left renal Ewing sarcoma following robotic-assisted retroperitoneal partial nephrectomy. Histopathology revealed a tumor composed of nests and lobules of monotonous tumor cells with round nuclei, indistinct nucleoli, and scanty cytoplasm, associated with a vascular-rich to hyalinized stroma and fibrillary neural matrix. Moderate nuclear pleomorphism, scattered necrosis, and pseudorosette formation were noted. Immunohistochemical studies demonstrated tumor cell positivity for CD99 and CD117. The patient was subjected to chemotherapy. Early diagnosis and multimodality treatment play an important role in improving survival rate.

## 1 Introduction

Approximately 90% of malignant renal neoplasms are renal cell carcinoma. Less than 1% of renal malignant neoplasms are renal sarcoma (1). Ewing sarcoma and peripheral primitive neuroectodermal tumors (PNETs) are a spectrum of neoplastic diseases known as Ewing sarcoma family tumors (ESFTs), which are a family of small round cell tumors that originate from the neural crest outside the central and sympathetic nervous system (2, 3). Adult primary renal Ewing sarcoma is extremely rare as most ESFTs are seen in the bones and soft tissues of children and adolescents. Most of the patients have poor prognosis and rapid clinical progression, including early metastasis (4), just like in prostate neuroendocrine tumors (5). The first renal Ewing sarcoma was reported in 1975 by Seemayer et al. (6), and since then, approximately 100 to 200 cases have been reported worldwide.

We reported a case of a 23-year-old male with primary left renal Ewing sarcoma treated with robotic-assisted retroperitoneal partial nephrectomy, which is one of the few partial nephrectomy treatments for Ewing sarcoma to date (7–11) (**Table 1**). We used three separate databases, namely, PubMed, Scopus, and Web of Science to search for articles with terms “renal Ewing sarcoma” and “partial nephrectomy.” Cases of renal Ewing sarcoma treated with either robotic or laparoscopic partial nephrectomy only were included. We reviewed publications from inception up to December 2024. Less than 10 cases were treated with partial nephrectomy worldwide.

**Table 1:** Demographic, tumor size, chemotherapy, local recurrence, and follow-up outcomes of primary renal Ewing sarcoma treated with partial nephrectomy.

Authors	Demographic	Tumor size	Tumor confined to kidney with negative margin	Adjuvant chemotherapy or radiotherapy (RT)	Local recurrence	Outcome
Tarek N (2020) (7)	41 years, male	–	Yes	Vincristine, doxorubicin, cyclophosphamide and actinomycin D × 1 cycle Noncompliance, no RT	Yes (9 months)	Alive with disease at 11 months post operation
Hakky TS (2013) (8)	33 years, male	5.1 × 4.8 × 3.3 cm	Yes	No	No	Alive without relapse at 12 months post operation
Kozel ZM (2020) (9)	14 years, female	5.6 cm	Yes	Vincristine, doxorubicin, and cyclophosphamide alternating with etoposide and ifosfamide, no RT	No	Alive without relapse at 3 months post operation
Suzuki I (2019) (10)	45 years, female	12 × 8 cm	Yes	No	No	Alive without relapse at 12 months post operation
Al-Gburi S (2024) (11)	19 years, female	3.5 × 3.3 × 2.0 cm	Yes	Vincristine, doxorubicin, and cyclophosphamide alternating with etoposide and ifosfamide, no RT	No	Alive without relapse at 6 months post operation

## 2 Case presentation

**Figure 1:**
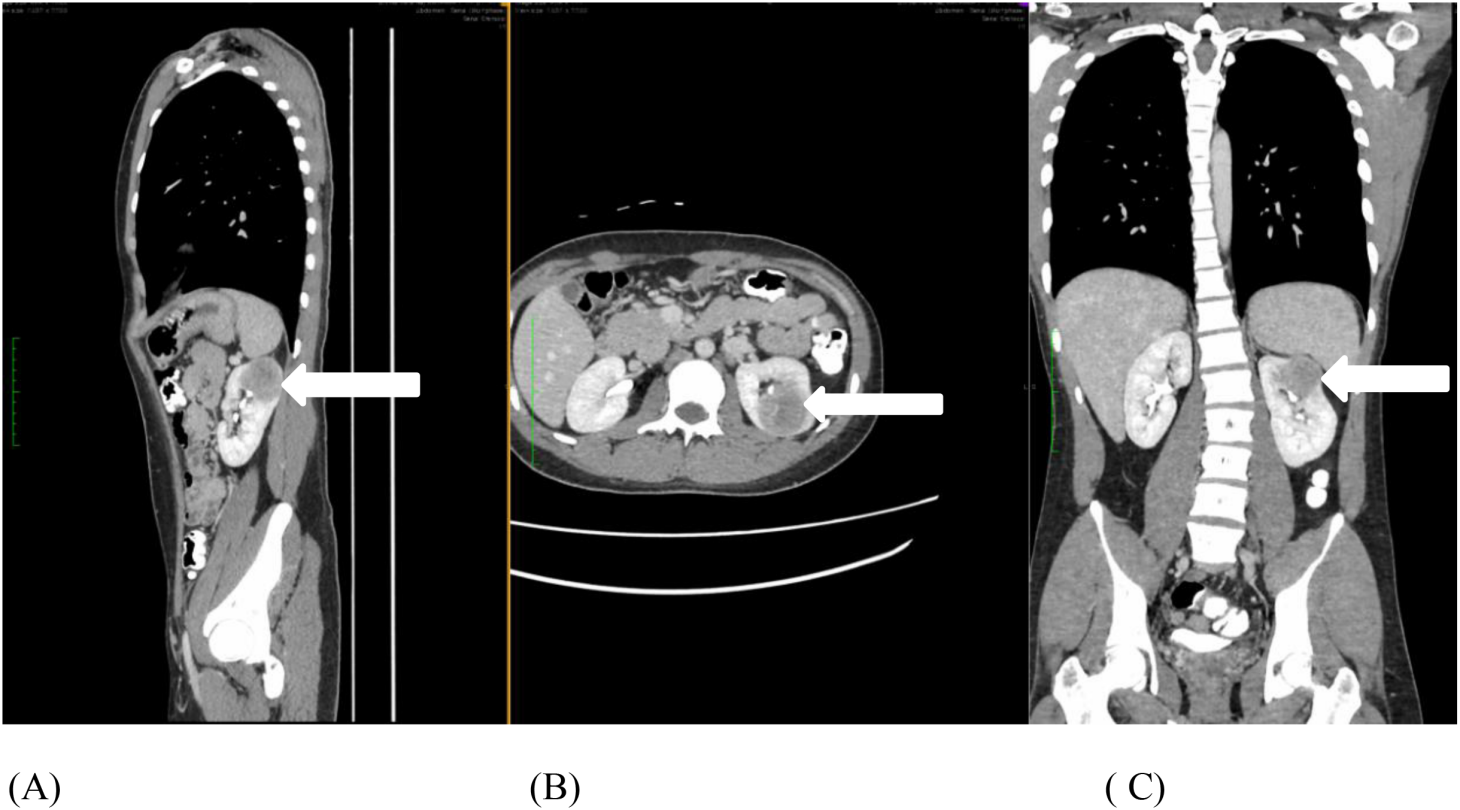
**(A-C)** Contrast-enhanced computed tomography of the abdomen and pelvis shows an enhanced, ill-defined heterogenous, exophytic lesion with central necrosis at the left upper pole of the kidney measuring 4.7 × 4.1 × 4.2 cm, with perinephric extension (white arrow).

A 23-year-old male with no other comorbidities presented with painless hematuria with blood clots and acute urinary retention. Clinically, no abdominal mass was palpable, and renal profile was normal. Contrast-enhanced computed tomography (CT) of the abdomen and pelvis revealed mildly enhancing, ill-defined, heterogeneous, exophytic lesion with an area of necrosis at the left upper pole of the kidney measuring 4.7 × 4.1 × 4.2 cm with perinephric extension (**Figures 1A–C**). CT thorax showed no lung metastasis or nodal involvement.

**Figure 2:**
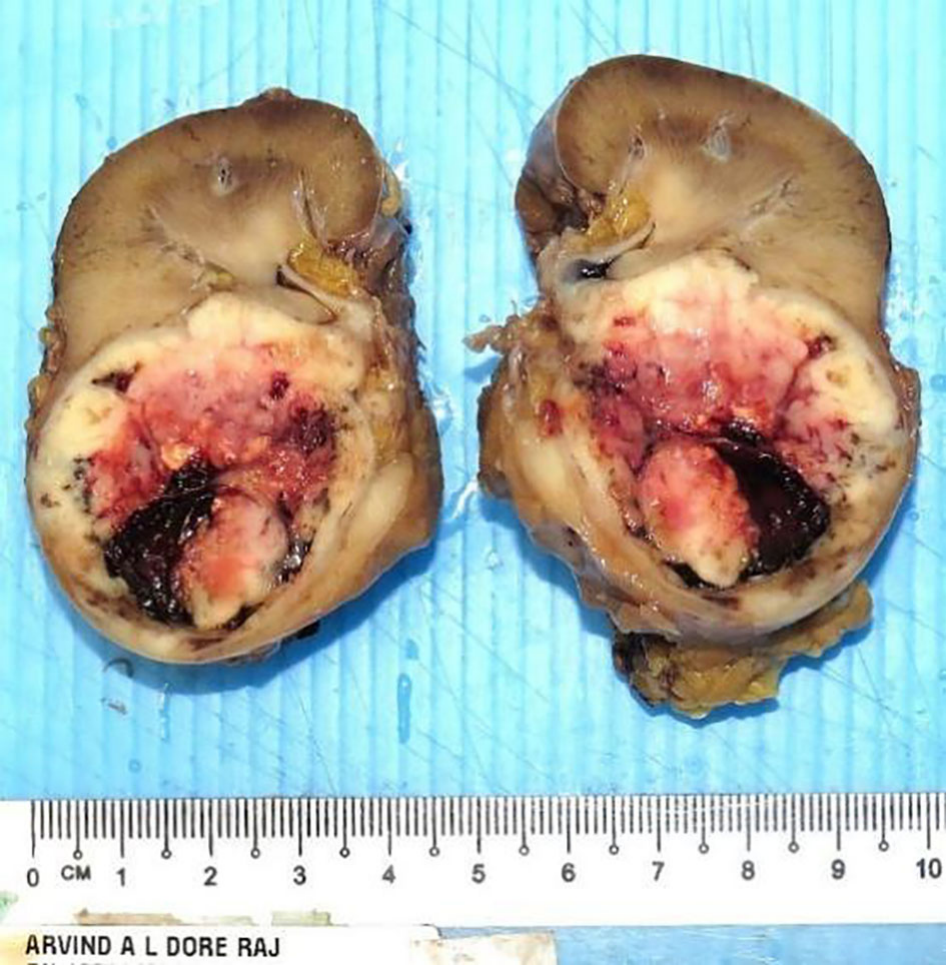
The left partial nephrectomy specimen showed a solid tumor with central necrosis measuring 45 × 40 × 38 mm and with clear surgery margin.

**Figure 3:**
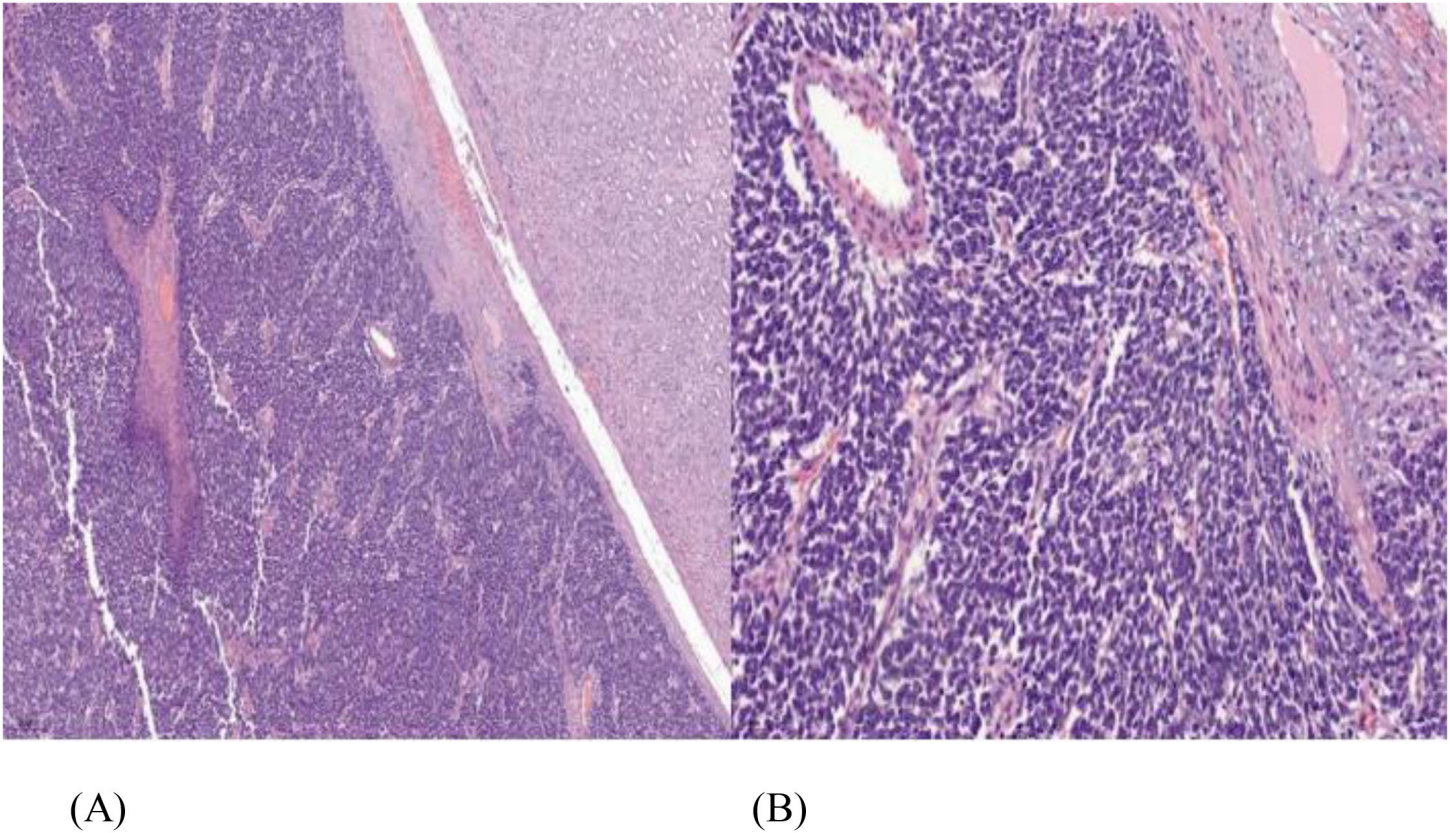
**(A)** Tumor comprised of loosely cohesive sheets and clusters of small round blue cells having mild-to-moderate eosinophilic cytoplasm, vesicular chromatin with small prominent nucleoli, hematoxylin and eosin staining (H&E) ×100. **(B)** Perivascular arrangement of tumor cells, (H&E) ×400.

He underwent a robotic-assisted retroperitoneal left partial nephrectomy. The renal nephrometry score for the tumor was 10×. The warm ischemic time was 18 min, and intraoperative blood loss was 150 ml. There was no significant postoperative creatinine change. He was discharged well on postoperative day 2. Histopathology reports a renal tumor size 45 × 40 × 38 mm with central necrosis (**Figure 2**). Microscopically, the tumor consisted of nests and lobules of monotonous tumor cells with round nuclei, indistinct nucleoli, and scanty cytoplasm, associated with a vascular-rich to hyalinized stroma and fibrillary neural matrix. Mitotic activity was estimated to be about 5 in 10 high-power fields. There was moderate nuclear pleomorphism with scattered necrosis. Pseudorosette was occasionally noted (**Figures 3A, B**). The surgical margin was clear of malignancy.

**Figure 4:**
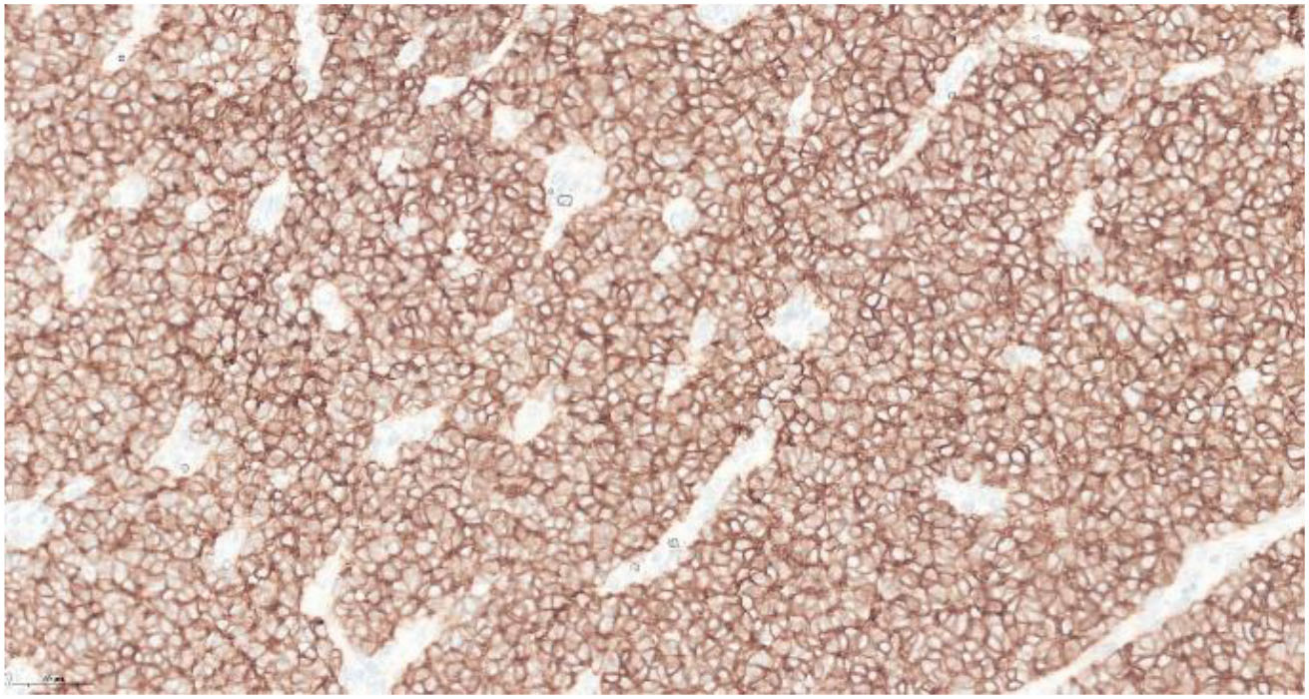
Immunohistochemical studies showed that the tumor cells were positive toward CD99.

Immunohistochemical studies showed that the tumor cells expressed positivity for CD99 and CD117 (**Figure 4**). The diagnosis of left renal Ewing sarcoma/PNET, pT1b pN0, was confirmed. A PET/CT scan was performed postoperatively and showed no evidence of nodal involvement or distant metastasis.

The patient was referred to an oncologist and planned for adjuvant chemotherapy as per Euro Ewing 2012 Arm B regime with VDC/IE for nine cycles, followed by reassessment with a PET/CT scan and potential radiotherapy to the tumor bed, and then consolidation chemotherapy with VC/IE for five cycles. However, after the third cycle of chemotherapy, the patient opted to stop the chemotherapy due to side effects, primarily tiredness. A dose reduction and supportive measures were offered to manage the symptoms, but the patient declined and chose to stop chemotherapy (**Table 2**).

**Table 2:** Management timeline.

Date	Timeline
Early September 2023	First presentation with painless hematuria and acute urinary retention
2 September 2023	Contrast-enhanced computed tomography (CT) of the abdomen and pelvis revealed mildly enhancing, ill-defined, heterogeneous, exophytic lesion with area of necrosis at the left upper pole of the kidney measuring 4.7 × 4.1 × 4.2 cm, with perinephric extension
23 September 2023	Robotic-assisted retroperitoneal left partial nephrectomy
5 October 2023	Histopathology: PNET/Ewing sarcoma, pT1b pN0
24 November 2023	Postoperative PET/CT scan showed no evidence of nodal or distant metastasis
5 February 2024	The patient was started on the Euro Ewing 2012 Arm B regime, which included VDC/IE for 9 cycles, followed by reassessment with a PET/CT scan and potential radiotherapy to the tumor bed, and then consolidation chemotherapy with VC/IE for 5 cycles
19 April 2024	Patient opted to discontinue chemotherapy after cycle 3 due to fatigue; a dose reduction was offered but declined

## 3 Discussion

Primary renal Ewing sarcoma/PNET is an extremely rare disease. It commonly affects the second and third decades of life with a mean age of 28 years. Male to female ratio is 3:1, with males predominantly affected (12). The pathogenesis of renal Ewing sarcoma remains unclear, although some literature suggests it may be related to chromosomal translocation involving the EWSR1 gene (13). Several theories propose the origin of PNET at peripheral sites, such as tumors from a neural ramification of the celiac plexus innervating the kidney, the presence of neural crest cells, or derivation from pluripotent germ cells or mesenchymal stem cells (14).

Clinical symptoms and signs are nonspecific; they may be asymptomatic or present as abdominal pain, palpable abdominal mass, or hematuria, which can occur in all renal tumors (15). Gross painless hematuria with blood clots in young adults should raise clinical suspicion for renal tumors, as seen in our case. There are no specific signs that can confirm the disease radiologically (16), although some reports mention CT findings that may aid in the diagnosis of renal Ewing sarcoma (17).

The morphologic similarity with other small round blue cell tumors makes the disease difficult to diagnose. The differential diagnosis of ES/PNET of kidney based on histology includes synovial sarcoma, the blastemal variant of Wilms tumor, small-cell carcinoma, lymphoblastic lymphoma, neuroblastoma, the solid variant of alveolar rhabdomyosarcoma, desmoplastic small round blue-cell tumor, small-cell variant of osteosarcoma, and clear-cell sarcoma of the kidney (18). ES/PNET typically shows classic pseudorosettes. The diagnosis of PNET is confirmed by immunohistochemistry (IHC) and molecular analysis. IHC markers, such as CD99, SYN, and FLI-1, can aid in the diagnosis (12), and molecular studies will show translocation between EWS (22q12) and FLI-1 (11q24) genes using reverse-transcriptase polymerase chain reaction (RT-PCR) or fluorescence *in situ* hybridization (FISH) technique (19). In our case, histopathological features and immunohistochemical findings were consistent with PNET/Ewing sarcoma. Molecular confirmation of EWSR1–FLI1 translocation was not performed due to the unavailability of RT-PCR and FISH testing in our center. While histopathological and immunohistochemical findings strongly supported the diagnosis of Ewing sarcoma/PNET, we acknowledge that molecular testing would have strengthened diagnostic certainty. A PET/CT scan was not performed prior to surgery as the diagnosis of Ewing sarcoma was not yet established. At presentation, the renal mass was suspected to be a localized malignant lesion without evidence of nodal or distant metastasis on contrast-enhanced CT of the thorax, abdomen, and pelvis. PET/CT scan is not routinely indicated preoperatively unless there is a high suspicion of metastatic disease. The PET/CT scan was subsequently performed postoperatively for staging and treatment planning after histopathological confirmation on Ewing sarcoma.

There is no standard treatment for Ewing sarcoma of the kidney. Treatment strategies for renal Ewing sarcoma typically include surgery, chemotherapy, and radiation (3). Nephrectomy is carried out for local control. Laparoscopic surgery is preferred over open surgery due to less morbidity and mortality. The standard surgery method is radical nephrectomy (17). In our case, we performed a robotic-assisted retroperitoneal partial nephrectomy for this young patient to preserve his left kidney. This is one of the few nephron-sparing surgeries for renal Ewing sarcoma reported to date (7–11). Partial nephrectomy was chosen over radical nephrectomy based on several considerations. The tumor was localized, small in size (4.7 × 4.1 × 4.2 cm), and confined to the upper pole of the kidney without involvement of the renal hilum or surrounding structures. Complete tumor excision with negative margins was achievable through a nephron-sparing approach. Additionally, the patient was a young adult with no significant comorbidities, and preserving renal function was prioritized to reduce long-term risk of chronic kidney disease. Robotic-assisted retroperitoneal partial nephrectomy provided excellent visualization and surgical precision making it a feasible and safe option for this selected case. The intraoperative surgical outcome and postoperative recovery were excellent. The surgical resection margin was clear, and no disease recurrence was detected after 1 year of follow-up.

Due to the aggressive nature of the disease and the potential for early metastases, multi-agent chemotherapy, based on classic bone Ewing sarcoma trial regimens, was initiated after surgery. The standard chemotherapy regimen includes a three-drug combination of doxorubicin, d-actinomycin, and vincristine, with alternating cycles of ifosfamide and etoposide. Radiotherapy is applied to treat local recurrences and residual tumors (20). A newer agent, like apatinib, an angiogenic drug, may be a therapeutic option for renal Ewing sarcoma, but more research is ongoing to confirm their benefits (21). In our case, Euro Ewing 2012 Arm B protocol was selected based on international standards for treating Ewing sarcoma, including extraosseous forms. This regime remains widely accepted due to the biological similarities across Ewing sarcoma sites. After the third cycle, the patient experienced significant fatigue. Supportive measures were started, and dose reduction was offered. However, the patient declined further treatment. This case highlights the real-world challenge of maintaining compliance with intensive chemotherapy regime in young adults and underscores the need for patient-centered approaches in rare malignancies without standard treatment pathways.

The 5-year disease-free survival rate for localized tumor is approximately 45%–55%, while the overall cure rate of metastatic disease is 20%, despite aggressive treatment (16).

Limitations and challenges in treating renal Ewing sarcoma/PNET include the lack of optimal and specific treatment protocols, primarily due to the rarity of the tumor and absence of large-scale clinical trials. Additionally, chemotherapy toxicity can significantly impact the patient’s quality of life and may lead to treatment discontinuation.

## 4 Conclusions

Renal Ewing sarcoma/PNET is very a rare and aggressive disease among young adults and carries a poor prognosis despite aggressive treatment. The clinical or radiological signs are non-specific, and require immunohistochemistry and molecular study for proper diagnosis. Early diagnosis and treatment play an important role in improving survival rates. Partial nephrectomy should be considered a surgical option for localized small renal Ewing sarcoma with negative surgical margins to preserve kidney function. Multimodal treatment, including surgery, chemotherapy, and/or radiotherapy, remains the treatment strategy.

## Data Availability

The original contributions presented in the study are included in the article/supplementary material. Further inquiries can be directed to the corresponding author.
